# Comparative mapping combined with homology-based cloning of the rice genome reveals candidate genes for grain zinc and iron concentration in maize

**DOI:** 10.1186/s12863-015-0176-1

**Published:** 2015-02-14

**Authors:** Tiantian Jin, Jingtang Chen, Liying Zhu, Yongfeng Zhao, Jinjie Guo, Yaqun Huang

**Affiliations:** Hebei Branch of Chinese National Maize Improvement Center, Agricultural University of Hebei, Baoding, People’s Republic of China

**Keywords:** Maize, Grain zinc and iron concentration, Meta-analysis, Comparative mapping, Ortho-mMQTL

## Abstract

**Background:**

Grain zinc and iron concentration is a complex trait that is controlled by quantitative trait loci (QTL) and is important for maintaining body health. Despite the substantial effort that has been put into identifying QTL for grain zinc and iron concentration, the integration of independent QTL is useful for understanding the genetic foundation of traits. The number of QTL for grain zinc and iron concentration is relatively low in a single species. Therefore, combined analysis of different genomes may help overcome this challenge.

**Results:**

As a continuation of our work on maize, meta-analysis of QTL for grain zinc and iron concentration in rice was performed to identify meta-QTL (MQTL). Based on MQTL in rice and maize, comparative mapping combined with homology-based cloning was performed to identify candidate genes for grain zinc and iron concentration in maize. In total, 22 MQTL in rice, 4 syntenic MQTL-related regions, and 3 MQTL-containing candidate genes in maize (ortho-mMQTL) were detected. Two maize orthologs of rice, GRMZM2G366919 and GRMZM2G178190, were characterized as natural resistance-associated macrophage protein (NRAMP) genes and considered to be candidate genes. Phylogenetic analysis of NRAMP genes among maize, rice, and *Arabidopsis thaliana* further demonstrated that they are likely responsible for the natural variation of maize grain zinc and iron concentration.

**Conclusions:**

Syntenic MQTL-related regions and ortho-mMQTL are prime areas for future investigation as well as for marker-assisted selection breeding programs. Furthermore, the combined method using the rice genome that was used in this study can shed light on other species and help direct future quantitative trait research. In conclusion, these results help elucidate the molecular mechanism that underlies grain zinc and iron concentration in maize.

## Background

Zinc and iron are essential micronutrients for all living organisms and play important roles in maintaining life. Zinc and iron deficiencies lead to serious diseases such as low immunity, stunted growth, and iron-deficiency anemia [1]. According to the World Health Organization (2002), zinc and iron deficiencies are the top-ranked health risk factors in developing countries [2]. It is estimated that about 30% and 60% of the world’s population suffers from diseases that are caused by zinc deficiency and iron deficiency, respectively [3-5]. Biofortification is the improvement of the concentration of essential minerals and vitamins in major staple crops through conventional plant breeding and modern biotechnology. This, combined with increasing the daily intake of such crops, has proven to be the most economical and sustainable approach for relieving micronutrient deficiency in the last decade worldwide [6-8].

Understanding the genetic mechanisms behind biofortified traits is the first step in biofortification. Over the past few years, some loci that are responsible for zinc and iron concentration-related traits have been detected through quantitative trait loci (QTL) mapping in various kinds of crops, in particular in grains of major staple foods such as rice (*Oryza sativa* L.) [9-16] and maize (*Zea mays* L.) [17-20], which have been shown to contain low levels of micronutrients. However, previous results that pertained to the genomic location, confidence intervals or total variance explained by QTL were inconsistent because of different genetic backgrounds, environments, and/or mapping methods. Therefore, comparative analysis of QTL that are revealed by independent experiments has become a popular research topic with substantial challenges.

Instead of manually compiling a large amount of QTL information, meta-analysis has been shown to be an effective tool for integrating and re-analyzing such data [21]. Using this method, the number of “real” QTL that were represented by QTL detected in different studies could be calculated and the refined position and the reduced confidence interval of the “real” QTL could be estimated. Meta-analysis has been used in different species to analyze a wide variety of traits, including grain yield and its related traits, flowering time and photoperiod sensitivity, drought tolerance, disease resistance, cold stress, nitrogen use efficiency, grain moisture, root and leaf architecture traits, fiber quality, oil content, and plant maturity traits [22-39]. We previously performed a meta-analysis on zinc and iron concentration in maize grains, and 10 meta-QTL (MQTL) were found [17]. MQTL could increase the accuracy and pace of genetic improvement of crops.

In the meta-analysis of grain zinc and iron concentration in maize, we found that the number of QTL is far less than those that are related to easily available traits such as plant height, because the phenotypic values of such traits are difficult to quantify. Fortunately, previous studies have shown that there is an extensive synteny between maize and rice genomes [40]. Therefore, combined analysis of the two species is an alternative way to use limited resources. Comparative mapping that uses common genetic markers to reveal synteny among different species is an ideal way to integrate the genetic information of independent genomes [41]. Conserved chromosome regions for important agronomic traits of maize and rice have been reported by comparative mapping of QTL in maize and rice [42,43]. Comparative mapping of MQTL with higher reliability could accurately uncover the conserved synteny for traits of interest. However, to our knowledge, no published study has compared MQTL.

In contrast with other visible traits, such as kernel length and width, only a few studies have been conducted on metabolic mechanisms of zinc or iron in maize, and only two gene families, nicotianamine synthase (NAS) and zinc-regulated transporter (ZRT), iron-regulated transporter (IRT)-like protein (ZIP), have been cloned and described [44,45]. Alternatively, the metabolic pathways of zinc and iron, from absorption to accumulation, have been extensively studied in rice, and many genes that are involved have been cloned and characterized, such as *OsNAS1-3*, *OsNAAT*, *OsDMAS1*, and *OsTOM1*, which participate in mobilization and absorption of cations around the rhizosphere [46-52]. Additionally, *OsYSL2*, *6*, *15*, *16*, *18*; *OsIRT1*, *2*, *OsZIP1*, *3*–*5*, *7a*, *8*; *OsNRAMP1*, *3*, *5*; *OsHAM2*, *3*, *5*, *9*; *OsMTP1*, *8.1*; *OsFRDL1*; *OsVIT1*, *2*; and OsTRO*2*, *3* are responsible for transportation and accumulation of cations in this species [53-91]. This gene information in rice, which is the model plant for other grasses, could be useful for identifying candidate genes for QTL or MQTL in maize [92].

Therefore, in this study, we combined comparative mapping with homology-based cloning using MQTL for grain zinc and iron concentration in maize (mMQTL) and rice (rMQTL) to predict candidate genes for maize. First, a meta-analysis on published QTL that control grain zinc and iron concentration-related traits in rice was performed to detect MQTL in this species. Then, these were compared with grain zinc and iron concentration MQTL in maize, which was previously reported by us through comparative mapping to identify the conserved synteny. Furthermore, positions of MQTL for maize zinc and iron concentration in grains and maize orthologs of rice zinc and iron metabolism-related genes were compared to reveal the relationship between these genes and the natural variation of this trait. Finally, phylogenetic degeneration of maize orthologs of the rice natural resistance-associated macrophage protein (NRAMP) gene family was elucidated to provide a foundation for further functional characterization.

## Results

### QTL meta-analysis for zinc and iron concentration in rice grains

**Figure 1:**
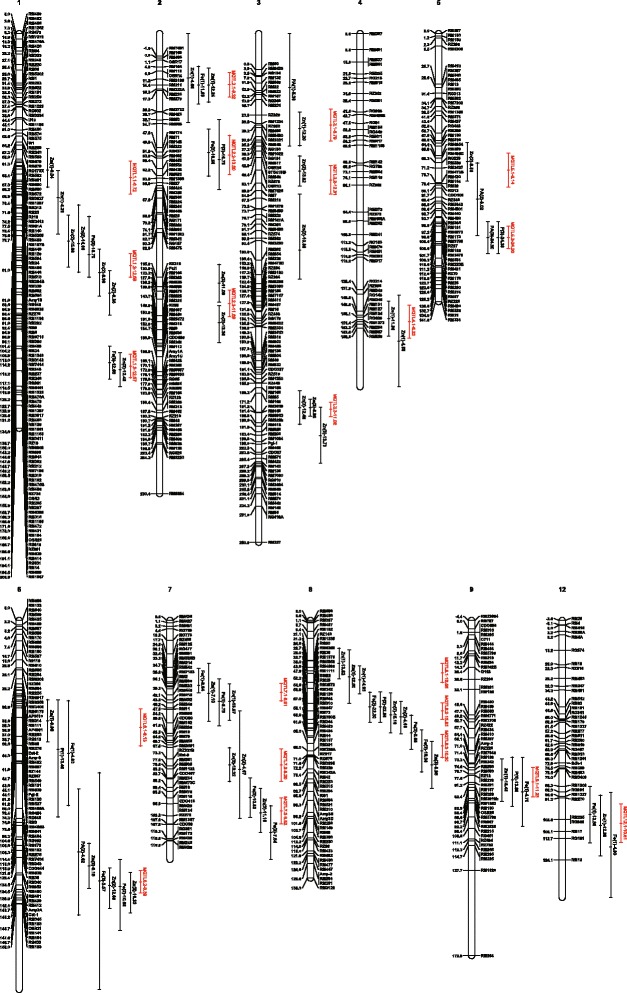
**Distribution of MQTL for grain zinc and iron concentration on rice chromosomes.** Vertical lines on the right of chromosomes indicate the confidence interval, and figures behind the name of initial QTL and MQTL connected by a dash indicate the phenotypic variance.

Meta-analysis was conducted to integrate and refine QTL for grain zinc and iron concentrations in rice when 74 of the 90 collected QTL were projected onto the consensus map. According to the definition of meta-analysis, chromosome regions that contained only one QTL were ignored during the analysis, which resulted in 63 QTL that were involved in integration. In total, 22 rMQTL were distributed across all rice chromosomes except chromosomes 10 and 11: three rMQTL on chromosomes 1, 2, 3, 7, and 8; two rMQTL on chromosomes 5 and 6; and one each on chromosomes 4, 9, and 12 (Figure 1).

Detailed information about rMQTL is provided in Table 1. The 22 rMQTL integrated two to six original QTL that were identified by independent experiments. The confidence intervals of the rMQTL, ranging from 7.68 cM (rMQTL3.3) to 20.66 cM (rMQTL2.2), were narrower than the mean confidence intervals of their respective original QTL. At three rMQTL, rMQTL3.3, rMQTL7.1, and rMQTL8.2, the confidence intervals were less than 10 cM. The phenotypic variance of the rMQTL varied from 6.72% (rMQTL1.1) to 24.30% (rMQTL5.2), and at 12 of the 22 rMQTL, the phenotypic variance was greater than 10%. In general, the rMQTL were represented by several original QTL that were associated with both grain zinc concentration and grain iron concentration.

**Table 1:** **MQTL for grain zinc and iron concentration in rice identified by meta-analysis**

**MQTL**	**Chr.**	**Position (cM)**	**QTL region**	**Closest maker**	**AIC**	**QTL model**	**No. of initial QTL**	**Mean phenotypic variance of the QTL**	**Mean initial QTL CI (cM)**	**MQTL CI (95%) (cM)**	**Physical distance (bp)**	**Related trait**
rMQTL1.1	1	76.17	RM600-RM5638	RM3412	97.82	4	2	6.72	27.08	17.47	9,464,568-20,936,057	Zn
rMQTL1.2	1	122.71	RM246-RM403	RM443			5	12.59	28.76	12.40	27,336,316-29,385,871	Zn, Fe
rMQTL1.3	1	175.87	RM1198-RM104	RM431			2	12.57	17.58	12.43	37,603,776-40,168,103	Zn, Fe
rMQTL2.1	2	14.12	RM110-RM3732	RM211	75.33	4	3	9.32	28.42	12.90	1,326,951-4,407,973	Zn, Fe
rMQTL2.2	2	51.26	RM555-RM550	RG437			2	13.60	30.95	20.66	4,305,688-12,464,529	Fe, P
rMQTL2.3	2	129.86	Pal1-RM599	RM263			2	11.69	19.69	13.92	24,973,386-27,115,300	Zn
rMQTL3.1	3	29.33	RM231-RM1022	RM489	83.49	4	2	8.79	38.55	16.97	2,454,089-7,233,990	Zn, PA
rMQTL3.2	3	58.28	RM546-RM218	RM7425			2	12.31	30.50	15.06	6,164,117-8,406,578	Zn
rMQTL3.3	3	179.73	RM168-RM5813	RM3919			3	11.02	18.73	7.68	28,098,585-30,981,264	Zn, Fe
rMQTL4.1	4	152.34	RM348-RM559	RM280	33.37	3	2	8.23	33.55	17.33	32,835,501-35,336,879	Zn
rMQTL5.1	5	72.11	RM516-RZ649	RM3437	29.84	2	2	8.41	29.33	17.91	8,304,202-19,608,342	Zn, PA
rMQTL5.2	5	107.85	RM3476-RM178	RM233B			2	24.30	16.91	11.96	23,906,571-25,164,524	PA
rMQTL6.1	6	56.64	RM539-RG424	RM527	109.12	4	3	9.13	46.55	19.61	8,170,581-19,814,539	Zn, Fe, P
rMQTL6.2	6	138.25	RM30-RM345	RM461			6	8.39	47.61	11.86	27,253,297-30,865,997	Zn, Fe, PA
rMQTL7.1	7	37.91	RM501-RM432	RM533	76.23	3	4	8.91	21.93	9.41	8,006,856-18,959,778	Zn, Fe
rMQTL7.2	7	76.47	RM3691-RM234	RM351			2	8.20	37.38	17.09	19,226,136-25,473,814	Zn
rMQTL7.3	7	100.73	RM478-RM1357	RZ978			3	9.85	24.15	13.62	25,950,515-28,852,240	Zn, Fe
rMQTL8.1	8	27.71	RM1235-RM1376	RM38	95.34	4	3	13.68	19.77	10.19	1,209,754-3,169,069	Zn
rMQTL8.2	8	47.86	RM4085-RM25	RM1111			4	15.85	17.51	8.60	4,450,273-4,378,594	Zn, Fe, P
rMQTL8.3	8	66.44	RM547-RM339	RM483			3	10.30	21.71	12.21	5,92,402-17,945,202	Zn, Fe
rMQTL9.1	9	81.06	RM242-RM5786	RM201	26.17	2	3	11.23	28.33	15.39	18,811,120-20,482,666	Zn, Fe, P
rMQTL12.1	12	104.78	RM270-RM12	RG958	40.16	3	3	10.51	39.17	20.32	25,002,547-26,988,436	Zn, Fe

AIC = Akaike Information Criterion, CI = confidence interval, cM = centiMorgan, bp = base pair.

### Syntenic MQTL-related regions between maize and rice

**Figure 2:**
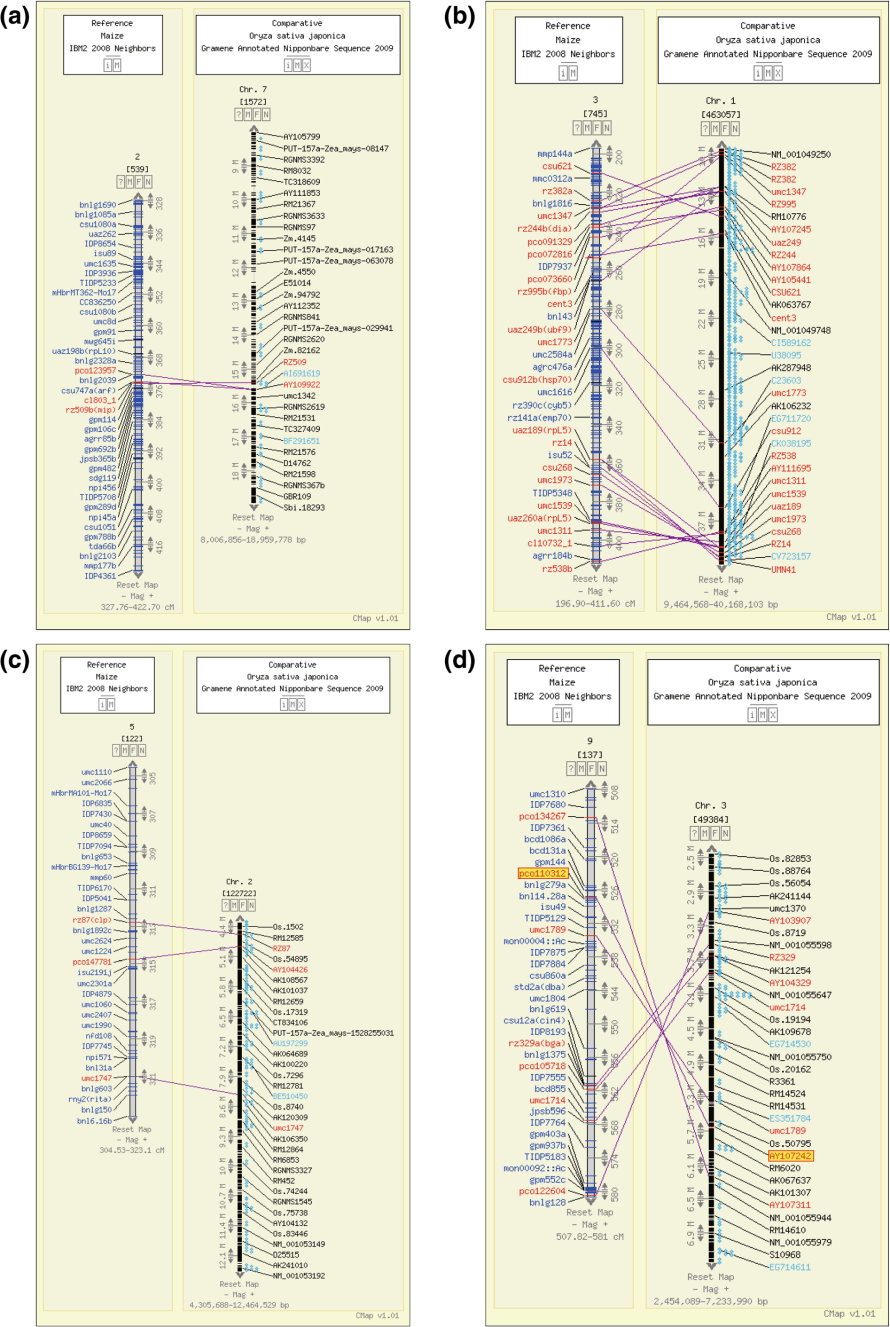
**Comparative maps between maize and rice.** The confidence interval of mMQTL2.1 was co-linear with the physical interval of rMQTL7.1 **(a)**; the confidence interval of mMQTL3 was co-linear with the physical intervals of rMQTL1.1 and rMQTL1.3 **(b)**; the confidence interval of mMQTL5 was co-linear with the physical interval of rMQTL2.2 **(c)**; the confidence interval of mMQTL9.2 was co-linear with the physical interval of rMQTL3.1 **(d)**.

Comparative mapping of MQTL for grain zinc and iron concentration between maize and rice was performed to study the conserved synteny for such traits when respective MQTL data were available through meta-analysis. In total, four syntenic MQTL-related regions with more than two common markers were received: mMQTL2.1 on maize chromosome 2 was co-linear with rMQTL7.1 on rice chromosome 7 (Figure 2a), mMQTL3 on maize chromosome 3 was co-linear with rMQTL1.1 and rMQTL1.3 on rice chromosome 1 (Figure 2b), mMQTL5 on maize chromosome 5 was co-linear with rMQTL2.2 on rice chromosome 2 (Figure 2c), and mMQTL9.2 on maize chromosome 9 was co-linear with rMQTL3.1 on rice chromosome 3 (Figure 2d).

Extensive database searching for common markers that were associated with maize and rice MQTL maps was carried out to seek the functional annotation information. An overgo probe, pco110312/AY107242, which is located in the intervals of mMQTL9.2 and rMQTL3.1, was able to anchor on the following metal transport protein-coding genes: GRMZM2G178190 in maize and *OsNRAMP2*, which belongs to the NRAMP gene family in rice (Figure 2d). Sequence alignment indicated that the protein sequence of the two genes showed very high identity (92%). Other common markers, however, had no functional information that was related to the target trait we studied.

### Characterization of the ortho-mMQTL

A total of 38 maize orthologs of rice zinc and iron metabolism-related genes were obtained through a homology-based cloning method, and their detailed information is listed in Table 2. After comparing the positions of mMQTL and maize orthologs of well-characterized rice genes, three ortho-mMQTLs that contained orthologs were discovered. The genomic region of ortho-mMQTL2.1 possessed the following maize orthologs: GRMZM2G085833 of the rice-cloned gene, *OsYSL6*, which belongs to the yellow stripe1-like (YSL) gene family; GRMZM2G366919 of the rice-cloned gene, *OsNRAMP1*, which belongs to the NRAMP gene family; and GRMZM2G175576 of the rice clone-gene, *OsHMA3*, which belongs to the heavy metal ATPase (HMA) gene family. The genomic region of ortho-mMQTL3 possessed the following maize orthologs: GRMZM2G063306 (*ZmTOM1*) of the rice-cloned gene *OsTOM1* and GRMZM2G057413 of the rice-cloned gene *OsIRO2*, which is a basic helix-loop-helix transcription factor. Additionally, the genomic region of ortho-mMQTL10 that possessed the maize ortholog GRMZM2G026391 of the rice-cloned gene *OsYSL16* also belonged to the rice YSL gene family.

**Table 2:** **Maize orthologs of rice well-characterized genes related to zinc and iron metabolism**

**References**	**Rice genes**	**Accession numbers**	**Main tissue expression**	**Gene products**	**Maize orthologs**
		(GenBank/TIGR)			(ID/Gene name/mMQTL)
[46]	*OsNAS1;*	AB021746/LOC_Os03g19427;	Leaves(Zn/Fe), Seeds(Zn/Fe)	Nicotianamine synthase	GRMZM2G030036/*ZmNAS2*;
[47]	*OsNAS2*	AB023818/LOC_Os03g19420	Roots(Fe), Shoots(Fe),		GRMZM2G034956/*ZmNAS1*;
			Leaves(Fe), Seeds(Fe)		GRMZM2G124785/*ZmNAS2;2*;
					GRMZM2G312481/*ZmNAS1;2*;
					GRMZM2G385200/*ZmNAS1*;
					GRMZM2G704488/*ZmNAS6;1*;
					AC233955.1_FGT003/*ZmNAS6;2*
[48]	*OsNAS3*	AB023819/LOC_Os07g48980	Roots(Zn/Fe), Shoots(Zn/Fe),	Nicotianamine synthase	GRMZM2G050108/*ZmNAS5*;
			Seeds(Zn/Fe/Cu)		GRMZM2G478568/*ZmNAS3*
[49]	*OsNAAT1*	AB206814/LOC_Os02g20360	Roots(Fe/Zn/Cd), Shoots(Fe/Zn/Cd),	Nicotianamine aminotransferase	GRMZM2G096958/*ZmNAAT1*;
[50]			Seeds(Fe)		GRMZM2G412604
[51]	*OsDMAS1*	AB269906/LOC_Os03g13390	Roots(Fe), Shoots(Fe)	Deoxymugineic acid synthase	GRMZM2G060952/*ZmDMAS1*
[52]	*OsTOM1*	AK069533/LOC_Os11g04020	Roots(Fe), Shoots(Fe), Seeds(Zn/Fe/Cu)	DMA efflux transporter	**GRMZM2G063306/** ***ZmTOM1*** **/mMQTL3**
[53,54]	*OsYSL2*	AB126253/LOC_Os02g43370	Roots(Fe), Shoots(Fe/Mn), Seed(Fe/Mn)	Iron-phytosiderophore transporter	n.a.
[55]	*OsYSL6*	AB190916/LOC_Os04g32050	Leaves(Mn)	Iron-phytosiderophore transporter	**GRMZM2G085833/mMQTL2.1**
[56,57]	*OsYSL15*	AB190923/LOC_Os02g43410	Roots(Fe), Shoots(Fe),	Iron-phytosiderophore transporter	GRMZM2G156599/*ZmYS1*
			Leaves(Fe), Seed(Fe)		
[58,59]	*OsYSL16*	AB190924/LOC_Os04g45900	Shoots(Fe), Leaves(Fe)	Iron-phytosiderophore transporter	**GRMZM2G026391/mMQTL10**
[60]	*OsYSL18*	AB190926/LOC_Os01g61390	Roots(Fe), Leaves(Fe), Flower(Fe)	Iron-phytosiderophore transporter	GRMZM2G004440
[61,62]	*OsIRT1*	AB070226/LOC_Os03g46470	Roots(Zn/Fe), Shoots(Zn/Fe), Seeds(Zn/Fe)	Metal ion transporter	GRMZM2G118821/*ZmIRT1*
[63]	*OsIRT2*	AB126086/LOC_Os03g46454	Root(Fe)	Metal ion transporter	n.a.
[64]	*OsZIP1*	AY302058/LOC_Os01g74110	Root(Zn)	Zinc/iron transporter	n.a.
[64]	*OsZIP3*	AY323915/LOC_Os04g52310	Roots(Zn), Leaves(Zn)	Zinc/iron transporter	GRMZM2G045849/*ZmZIP3*
[65,66]	*OsZIP4*	AB126089/LOC_Os08g10630	Roots(Zn), Shoots(Zn), Seeds(Zn)	Zinc/iron transporter	GRMZM2G111300/*ZmZIP4*
[67]	*OsZIP5*	AB126087/LOC_Os05g39560	Roots(Zn), Shoots(Zn)	Zinc/iron transporter	GRMZM2G047762
			Leaves(Zn), Seeds(Zn)		
[68]	*OsZIP7a*	AY275180/LOC_Os05g10940	Root(Fe)	Zinc/iron transporter	GRMZM2G015955/*ZmZIP7*
[68,69]	*OsZIP8*	AY327038/LOC_Os07g12890	Roots(Zn), Shoots(Zn), Seeds(Zn)	Zinc/iron transporter	GRMZM2G093276/*ZmZIP8*
[70,71]	***OsNRAMP1/rMQTL7.1***	AK103557/LOC_Os07g15460	Roots(Cd/Al), Leaves(Fe/Cd)	Natural resistance associated macrophage protein	**GRMZM2G366919/mMQTL2.1**
[72]	*OsNRAMP3*	AK070574/LOC_Os06g46310	Roots(Mn), Shoot(Mn), Leaves(Mn)	Natural resistance associated macrophage protein	GRMZM2G069198
[73,74]	*OsNRAMP5*	AK070788/LOC_Os07g15370	Roots(Fe/Mn/Cd), Shoots(Fe/Mn/Cd), Seeds(Mn/Cd)	Natural resistance associated macrophage protein	GRMZM2G147560
[75-77]	*OsHMA2*	AK107235/LOC_Os06g48720	Roots(Zn), Shoots(Zn/Cd), Leaves(Zn/Cd), Seeds(Zn/Cd)	P_1B_-type heavy-metal ATPases	GRMZM2G099191
[78,79]	*OsHMA3*	AB557931/LOC_Os07g12900	Roots(Cd), Shoot(Cd), Seeds(Cd)	P_1B_-type heavy-metal ATPases	**GRMZM2G175576/mMQTL2.1**
[80]	*OsHMA5*	AK063759/LOC_Os04g46940	Roots(Cu),Shoots(Cu), Seeds(Cu)	P_1B_-type heavy-metal ATPases	GRMZM2G143512
					GRMZM2G144083
[81]	*OsHMA9*	AK241795/LOC_Os06g45500	Roots(Pb), Shoots(Zn/Cu/Cd/Pb)	P_1B_-type heavy-metal ATPases	GRMZM2G010152
[82,83]	*OsMTP1*	AK100735/LOC_Os05g03780	Roots(Zn/Cd/Ni), Leaves(Zn/Cd), Seeds(Zn/Cd)	Cation diffusion facilitator	GRMZM2G477741
[84]	*OsMTP8.1*	AK065961/LOC_Os03g12530	Roots(Mn), Shoot(Mn)	Cation diffusion facilitator	GRMZM2G118497
[85]	*OsFRDL1*	AK101556/LOC_Os03g11734	Roots(Fe), Shoots(Fe)	MATE efflux family protein	GRMZM2G163154
[86]	*OsVIT1*	AK059730/LOC_Os04g38940	Leaves(Zn/Fe), Seeds(Zn/Fe)	Vacuolar membrane transporters	GRMZM2G107306
[86,87]	*OsVIT2*	AK071589/LOC_Os09g23300	Shoots(Zn/Fe/Cu/Mn), Leaves(Zn/Fe), Seeds(Zn/Fe)	Vacuolar membrane transporters	GRMZM2G074672
[88-90]	*OsIRO2*	AK073385/LOC_Os01g72370	Roots(Fe), Shoots(Fe/Mn), Leaves(Fe), Seeds(Fe/Mn)	bHLH transcription factor	**GRMZM2G057413/mMQTL3**
[91]	*OsIRO3*	AK061515/LOC_Os03g26210	Roots(Fe), Shoots(Fe)	bHLH transcription factor	GRMZM2G350312

Maize orthologs located in mMQTL regions are emphasized in bold.

In comparison, ortho-mMQTL2.1 has attracted a substantial amount of attention because it is a “hot spot” of maize orthologs of rice genes and also because of the synteny between mMQTL2.1 and rMQTL7.1 that was revealed by comparative mapping. Additionally, the rice gene *OsNRAMP1*, which is located in the interval of MQTL7.1, is homologous with GRMZM2G366919, which is a maize ortholog that is located in the region of mMQTL2.1. Therefore, mMQTL2.1 and rMQTL7.1 were co-linear and contained a pair of homologous genes, GRMZM2G366919/*OsNRAMP1*.

### Identification and analysis of maize NRAMP genes

Because of the homology of the two pairs of genes in maize and rice, GRMZM2G366919/*OsNRAMP1* and GRMZM2G178190/*OsNRAMP2*, and their significant association with the natural variance of grain zinc and iron concentration, members of the NRAMP gene family in maize were searched, and a phylogenetic tree was built to elucidate the relationship between the gene function and genome evolution as well as provide a foundation for further functional characterization.

**Figure 3:**
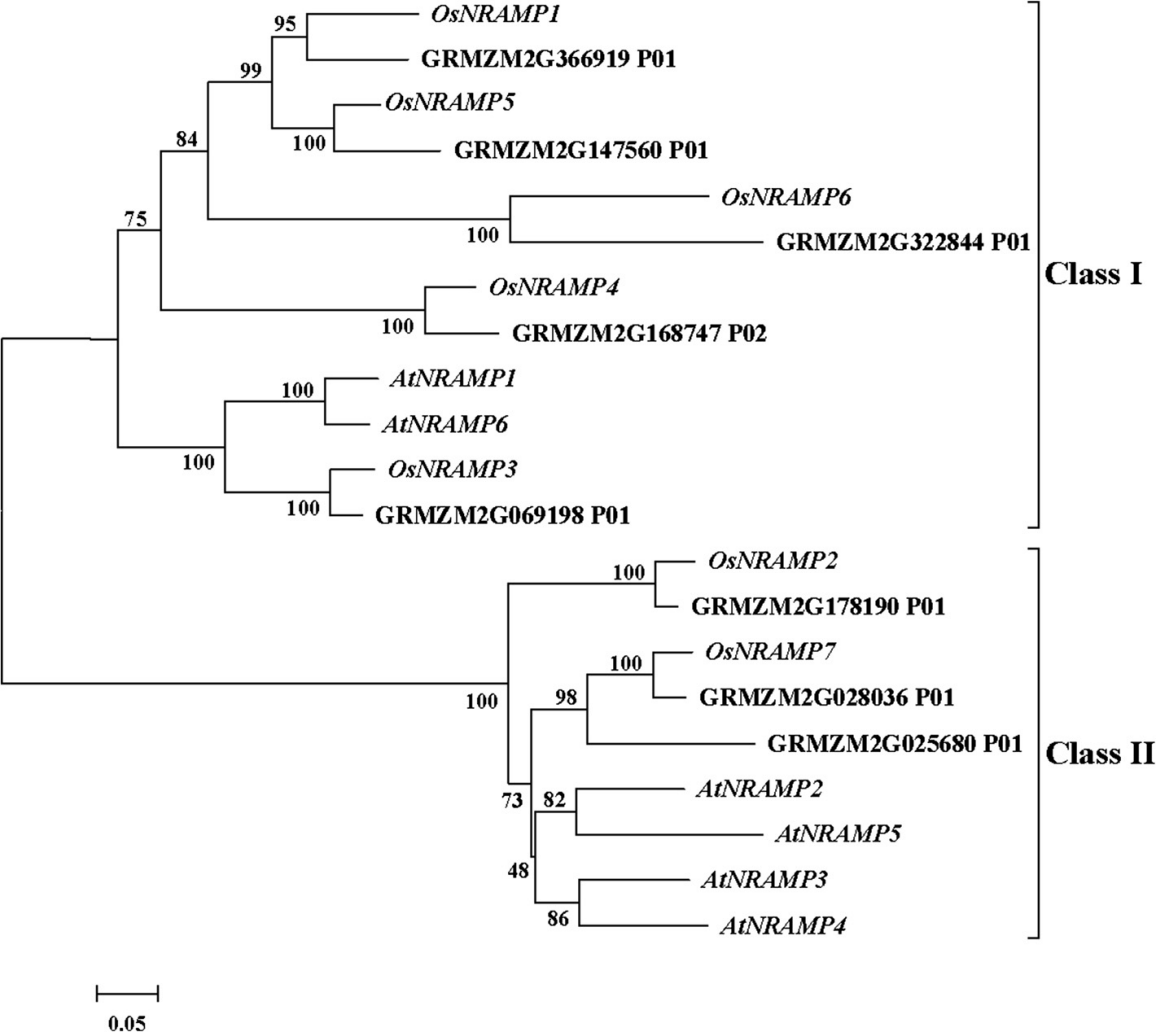
**Phylogenetic relationships of the NRAMP members among maize, rice and** ***Arabidopsis thaliana.*** The tree was built with the amino acid sequences of NRAMP proteins from maize, rice (Os) and *Arabidopsis thaliana* (At) using the neighbor-joining method in MEGA v4.0 software. The accession numbers were: *AtNRAMP1* (At1g80830), *AtNRAMP2* (At1g47240), *AtNRAMP3* (At2g23150), *AtNRAMP4* (At5g67330), *AtNRAMP5* (At4g18790), *AtNRAMP6* (At1g15960), *OsNRAMP1* (LOC_Os07g15460), *OsNRAMP2* (LOC_Os03g11010), *OsNRAMP3* (LOC_Os06g46310), *OsNRAMP4* (LOC_Os02g03900), *OsNRAMP5* (LOC_Os07g15370), *OsNRAMP6* (LOC_Os01g31870), *OsNRAMP7* (LOC_Os12g39180).

Eight putative genes in the maize genome were identified using reported NRAMP proteins from *Arabidopsis thaliana* as database queries. The phylogenetic tree was then constructed when all of the maize NRAMP proteins were aligned with the *A. thaliana* and rice NRAMP proteins (Figure 3). The NRAMP genes were divided into two groups based on the phylogenetic relationships: Class I and Class II. Most of the maize (5 of 8) and rice (5 of 7) NRAMP genes were categorized into Class I. A few were categorized into Class II. For *A. thaliana*, a model eudicot, the opposite occurred. A phylogenetic analysis showed that GRMZM2G366919, which is closely related to *OsNRAMP1*, was placed into Class I, a class which also contained *AtNRAMP1, 6* and *OsNRAMP3, 4, 5, 6*. GRMZM2G178190, which is closely related to *OsNRAMP2*, was categorized into Class II, a class which also contained *AtNRAMP2, 3, 4, 5* and *OsNRAMP2, 7*.

## Discussion

### Meta-analysis for QTL integration

Grain zinc and iron concentration is a polygenic trait that is controlled by QTL. Quantifying this trait is time consuming, laborious, and expensive. Consequently, comparing QTL for traits that are identified by independent experiments is important. Meta-analysis has been shown to be effective for QTL integration, and consensus QTL, with more accurate positions and reduced confidence intervals, could be provided [23]. In this study, a total of 90 collected QTL for zinc and iron concentration in rice grains were integrated into 22 rMQTL with a 65% decrease in total QTL through meta-analysis. The confidence intervals of rMQTL decreased by 29% to 75% compared with corresponding mean confidence intervals of several initial QTL.

We have previously conducted a meta-analysis on this trait in maize. Similarly, the 64% decrease in total QTL and 29% to 83% decreases in confidence intervals of mMQTL were achieved [19]. The genetic and physical intervals of MQTL could even be reduced to approximately 2 cM and 500 kb, respectively, in the meta-analysis for grain yield QTL that were detected in grasses during agricultural drought [25]. Therefore, meta-analysis can effectively synthesize and refine multiple independent QTL that are detected under different genetic backgrounds, population types and sizes, mapping statistics, and even phenotypic methodologies. The precise position and reduced confidence intervals for MQTL will pave the way for further QTL fine mapping and map-based cloning.

In addition to integrating independent QTL, meta-analyses can also reveal the genetic correlations among different traits. In a meta-analysis of QTL for leaf architecture traits, four MQTL were identified for three or four traits [38]. In accordance with previous knowledge that plant digestibility is associated with cell wall composition in maize, meta-analysis of QTL for the two traits showed that 42% of MQTL for digestibility had confidence intervals that overlapped with MQTL for cell wall composition traits [93].

In the current study, most rMQTL for grain zinc and iron concentration in rice were found to include QTL of both traits. Furthermore, in maize, meta-analysis of QTL for the same traits also showed that 8 of 10 mMQTL involved the two QTL traits, simultaneously. The correlation of grain zinc concentration and grain iron concentration at the molecular level strongly indicates that the variation loci responsible for the two traits were co-localized in both maize and rice genomes, or even in other species. MQTL for multiple traits could facilitate the genetic improvement through marker-assisted selection breeding programs.

### Synteny of grain zinc and iron concentration between maize and rice

There is a well-known evolutionary relationship between maize and rice, which are two major Gramineae species. Comparative mapping of QTL is useful for revealing the syntenic relationships of target traits among different species. For example, comparative analysis revealed that QTL for important agronomic traits, including plant height, number of rows, and kernels per row, are extensively conserved in the syntenic genomic regions of maize and rice [44,45]. In this study, comparative mapping for MQTL that control grain zinc and iron concentration in maize and rice was performed, and four syntenic MQTL-related regions were found. Moreover, the pco110312 overgo probe linked mMQTL9.2 and rMQTL3.1, which are syntenic MQTL-related regions, can anchor onto metal transport protein-coding genes, GRMZM2G178190 and *OsNRAMP2*. Although no candidate gene was found in other syntenic MQTL-related regions, they provided a foundation for future candidate gene mining. Therefore, the results here illustrate that grain zinc and iron concentration are syntenic between maize and rice, and the syntenic MQTL-related regions are reliable for subsequent analysis.

Based on the comparative mapping results, the four syntenic MQTL-related regions discussed aboved all had relatively broad intervals, which indicating that it was easier to find the respective syntenic region in the other species when MQTL had large confidence intervals. These results could provide a foundation for future research on these MQTL. Because of the narrowed intervals, no syntenic regions were found in MQTL with small confidence intervals. However, some of those MQTL, such as mMQTL2.2 and rMQTL8.2, integrated multiple initial QTL and explained a large percent of phenotypic variation, could provide insight into detection of new functional genes that underlie grain zinc and iron concentration.

### Homology-based cloning of maize grain zinc and iron concentration-related genes

Only one candidate gene for grain zinc and iron concentration in maize was discovered in the four conserved genomic regions. Only one gene may have been discovered because the online comparison is limited by the data that are available in public databases. Nevertheless, some rice functionally-characterized zinc and iron metabolism-related genes can be used for homology-based cloning of maize genes. Therefore, the positions of mMQTL and maize orthologs of rice-cloned genes were compared to validate the function of those genes for grain zinc and iron concentration variation in maize. Three ortho-mMQTLs with candidate genes were found. In particular, ortho-mMQTL2.1, which contained GRMZM2G366919, was co-linear with rMQTL7.1, and the corresponding orthologous gene, *OsNRAMP1*, was located in the genomic region of rMQTL7.1.

In a similar comparison of locations between maize orthologs of rice yield genes and MQTL, three candidate loci for maize yield were successfully predicted [94]. By mapping maize orthologs of rice- and *A. thaliana-*cloned genes that are associated with leaf architecture traits on the consensus map before OTL meta-analysis, Ku *et al*. also discovered candidate genes for the traits that they studied [38]. Overall, functionally-characterized genes in rice, which is a model species of Gramineae, could be used to identify and analyze candidate genes in maize or other grasses.

### Characterization of the maize NRAMP gene family

NRAMP was first identified in rat macrophages as a resistance gene to intracellular pathogens that transport iron [95]. Subsequently, many homologues of rat NRAMP that transport various cations, not merely iron, were characterized in plants. NRAMP genes are, in general, associated with membrane-spanning proteins [96] and widely distributed both in graminaceous and non-graminaceous species. To date, a total of 6 and at least 7 NRAMP genes have been cloned and some of them have been well-characterized in *A. thaliana* and rice, respectively.

In this study, two candidate genes in maize, GRMZM2G366919 and GRMZM2G178190, were identified as being associated with the natural variation of grain zinc and iron concentration through comparative mapping of MQTL combined with a homology-based cloning method with the rice genome. Based on their homology with rice NRAMP genes, members of the maize NRAMP gene family were mined, and a phylogenetic analysis of NRAMP genes in *A. thaliana*, rice, and maize was carried out to determine the evolutionary relationships among the genes. GRMZM2G366919, which is included in Class I, is closely related to *OsNRAMP1*, which participates in the control of iron, cadmium, and aluminum homoeostasis in rice [72,73,97]. *OsNRAMP5*, similar to *OsNRAMP1*, is relatively closely related to GRMZM2G366919, which contributes to iron, cadmium, and manganese transport in rice [75,76,98]. Interestingly, *AtNRAMP1*, which is also contained in Class I, is an iron transporter in *A. thaliana* and is able to rescue both low and high iron-sensitive phenotypes of the yeast mutant *fet3fet4* [97]. GRMZM2G178190 and *OsNRAMP2* are classified into Class II and are most closely related to each other, and *OsNRAMP2* was predicted to be a metal homeostasis gene in rice, although its specific function has not yet been clarified [99,100]. It is also worth noting that, with in Class II, *AtNRAMP3* and *AtNRAMP4* are capable of transporting iron, cadmium, and manganese in *A. thaliana* [101,102], and *AtNRAMP3* disruption can increase the accumulation of zinc in roots under iron starvation [103]. Therefore, the phylogenetic analysis demonstrated that GRMZM2G366919 and GRMZM2G178190 might be responsible for zinc and iron metabolism in maize and might be more likely to regulate their accumulation in grains.

### Implications for quantitative trait genetic research

Zinc and iron concentration in grains is undoubtedly a complex agronomic trait and plays a vital role in maintaining human health. However, the genetic basis of grain zinc and iron concentration remains obscure, despite many studies that have been conducted to identify QTL or genes that underlie this trait. We performed meta-analysis of QTL for grain zinc and iron concentration in rice in the present study and maize in a previous study [17] to detect the respective MQTL. However, in this study, to eliminate the limitation imposed by the lack of genetic information from one genome, we combined comparative mapping and homology-based cloning with the rice genome.

The MQTL allowed mining of candidate genes for grain zinc and iron concentration in maize. Two maize orthologs of rice NRAMP genes validated the power and effectiveness of the combined method that we adopted. Additionally, the combined method, as well as the well-studied rice genome employed here, can be extended to research on other species or complex traits.

## Conclusion

Enriching the concentration of zinc and iron in edible parts of major crops is an effective way to relieve malnutrition that is caused by zinc and iron deficiencies, and determining the molecular basis of grain zinc and iron concentration is a prerequisite for biofortification. Meta-analysis of QTL for very complicated traits such as grain zinc and iron concentration is important and useful. MQTL that are the integration of multiple independent QTL, with more precise locations and reduced confidence intervals, are useful for facilitating subsequent research. Candidate genes that were retrieved from the combination of comparative mapping of MQTL and homology-based cloning techniques could be used to reveal the molecular mechanisms that underlie zinc and iron concentration in maize grains. Syntenic MQTL-related regions and ortho-mMQTLs that contain candidate genes could be used for further fine mapping and map-based cloning.

## Methods

### QTL meta-analysis

Three steps were required for conducting the meta-analysis to identify MQTL. First, a bibliographic review on the mapping of QTL for zinc and iron concentration-related traits in rice grains was performed. The QTL information was collected from published reports including journal articles and dissertations. In all, eight reports involving nine mapping populations and 90 QTL were compiled. The details of those studies are provided in Table 3. Second, a consensus map that was integrated from multiple independent genetic linkage maps was built. The rice genetic linkage map Cornell SSR 2001 was selected as a reference map on which the maps of 8 studies were projected to develop the consensus map [104]. Third, a meta-analysis of QTL clusters on each chromosome was launched to detect MQTL. The modified Akaike’s information criterion (AIC) was used to select the QTL model; the model with the lowest AIC value was chosen as the best model, indicating the most likely number of “real” QTL on each chromosome [21]. Biomercator v2.1 was used to construct the consensus map with the “map projection” function and to conduct meta-analysis with the “meta-analysis” function [105].

**Table 3:** **Bibliography of QTL research for grain zinc and iron concentration in rice used in this study**

**QTL studies**	**Parents**	**Population types**	**Population size**	**No. of environments**	**Software and methods**	**No. of QTLs**	**Related traits**
[9]	IR64/Azucena	DH	129	1	QTL Cartographer v2.5 Composite interval mapping	8	Zn,Fe,PA
[10]	LPA/Zhonghua 11	F_2_	172	1	R/qtlbim Bayesian model selection	3	PA
[11]	Fengxinhongmi/Minghui 100	F_2_	145	1	QTL Cartographer v2.5 Composite interval mapping	3	Zn
[12]	Chunjiang 06/TN1	DH	120	2	Mapmaker/QTL v1.1 Interval Mapping	14	Zn,Fe,P
[13]	Hongxiang 1/Song 98-131	F_2:3_	140	1	QTL IciMapping v3. 1 Inclusive Composite Interval Mapping	6	Zn,Fe
[14]	Longjin 1/Xiangruanmi 1578	F_2:3_	196	1	QTLCartographer v2.0 Composite Interval Mapping	14	Zn,Fe,P
[15]	Chuanxiang 29B/ Lemont	RIL	184	2	QTL Cartographer v2.5 Composite Interval Mapping	8	Zn,Fe
[16]	Zhongguangxiang 1/IR75862	BC_1_F_7_	240	2	QTLMapper v1.0	14	Zn,Fe
	Ce 258/IR75862	BC_1_F_7_	240	2	Composite Interval Mapping	20	Zn,Fe

### MQTL comparative mapping

Integrated MQTL for grain zinc and iron concentration-related traits in rice were compared with MQTL for the same traits in maize. The CMap program on the Gramene (http://www.gramene.org/) was used to investigate the synteny of grain zinc and iron concentration in the two species. Maize was selected as the reference species using IBM2 2008 Neighbors as the reference map and then the rice physical map, Gramene Annotated Nipponbare Sequence 2009, was added as a comparative map with rMQTL anchored first. In this study, comparative maps with fewer than three common markers were discarded. To facilitate the description, MQTL for grain zinc and iron concentration in maize, which we have previously reported [17], were renamed (Table 4). Common markers that linked the two genomes were searched for (primarily in GeneBank, http://www.ncbi.nlm.nih.gov/genbank/, and Gramene) to identify their genomic annotation information.

**Table 4:** **Renamed maize MQTL with syntenic MQTL in rice**

**Renamed maize MQTL**	**Original maize MQTL**	**Maize bin**	**Position (cM)**	**Confidence interval (cM)**	**Physical distance (bp)**	**Rice syntenic MQTL**	**Rice chr.**
mMQTL2.1	MQTL1	2.04-2.07	377.1	327.8-422.7	55,353,009-193,786,994	MQTL7.1	7
mMQTL2.2	MQTL2	2.07	466.7	466.7-474.8	202,340,532-204,180,634	*n.a.*	*n.a.*
mMQTL2.3	MQTL3	2.08	573.9	557.3-589.1	214,654,418-220,845,300	*n.a.*	*n.a.*
mMQTL3	MQTL4	3.04-3.06	305.8	196.9-411.6	29,978,219-174,835,520	MQTL1.1, MQTL1.3	1
mMQTL4.1	MQTL5	4.06	354.1	349.6-367.3	153,770,346-163,275,597	*n.a.*	*n.a.*
mMQTL4.2	MQTL6	4.08	462.5	447.0-481.2	180,430,966-186,492,818	*n.a.*	*n.a.*
mMQTL5	MQTL7	5.04	312.8	304.5-323.1	84,815,350-150,635,401	MQTL2.2	2
mMQTL9.1	MQTL8	9.01	68.5	62.3-82.3	9,117,641-11,575,112	*n.a.*	*n.a.*
mMQTL9.2	MQTL9	9.06-9.07	554.4	507.8-581.0	146,944,409-151,490,783	MQTL3.1	3
mMQTL10	MQTL10	10.04	344.8	311.4-375.8	127,361,349-137,839,102	*n.a.*	*n.a.*

*n.a.* not available.

### Ortho-mMQTL mining

Detailed information on 33 cloned rice zinc or iron metabolism-related genes, including NAS, NAAT1, DMAS1, TOM1, YSL, ZIP, NRAMP, HMA, MTP, FRDL, VIT, and IRO was retrieved from the Rice Genome Annotation Project database (http://rice.plantbiology.msu.edu/). Maize orthologs of the 33 rice genes were identified by searching the databases of the Rice Genome Annotation Project, NCBI (http://www.ncbi.nlm.nih.gov/), B73 maize sequence (http://www.maizesequence.org) and Phytozome (http://phytozome.jgi.doe.gov/pz/portal.html) using the BLAST program. Their physical locations were identified using the maize genome browser, MaizeGDB (http://www.maizegdb.org/). Subsequently, the positions of mMQTL and maize orthologs were specifically compared to reveal the relationship between maize orthologs of rice zinc or iron-metabolism related genes and the natural variance of zinc and iron concentration in maize grains. In this study, mMQTL-possessing maize orthologs of rice zinc or iron metabolism-related genes were temporarily called ortho-mMQTL.

### Maize NRAMP genes identification and phylogenetic analysis

Members of the maize NRAMP gene family were identified using the BLASTP program in the Phytozome database by employing the protein sequence of previously identified *A. thaliana* NRAMP genes as queries. The threshold of e-value and identity for the BLASTP program were set at 1e-80 and >75%, respectively. In addition, protein motifs were searched for in the Pfam database (http://pfam.sanger.ac.uk) to confirm the candidate sequence that encodes NRAMP proteins. Multiple alignments of NRAMP proteins from maize, rice and *A. thaliana* were performed using the ClustalX program [106]. The phylogenetic tree was constructed using MEGA v4.0 software with the neighbor-joining (NJ) method and 1,000 bootstrap replicates [107].

### Supporting data

The phylogenetic tree of the present study is deposited in Treebase (http://purl.org/phylo/treebase/phylows/study/TB2:S17020?x-access-code=113cee34da6e7a2427055be64800c677&format=html).
